# Online Gaming Addiction and Basic Psychological Needs Among Adolescents: The Mediating Roles of Meaning in Life and Responsibility

**DOI:** 10.1007/s11469-022-00994-9

**Published:** 2023-01-10

**Authors:** Alican Kaya, Nuri Türk, Hasan Batmaz, Mark D. Griffiths

**Affiliations:** 1 Department of Guidance and Psychological Counselling, Ağrı İbrahim Çeçen University, Ağrı, Turkey; 2 Department of Guidance and Psychological Counselling, Siirt University, Siirt, Turkey; 3 Department of Guidance and Psychological Counselling, Sakarya University PhD Student, Sakarya, Turkey; 4 International Gaming Research Unit, Psychology Department, Nottingham Trent University, 50 Shakespeare Street, Nottingham, NG1 4FQ UK

**Keywords:** Basic psychological needs, Online gaming addiction, Responsibility, Meaning in life

## Abstract

Individuals whose basic needs are naturally satisfied are much less dependent on their environment and more autonomous. Basic psychological needs (i.e., the general motivators of human actions) are significant predictors of online gaming addiction. Moreover, it has been posited that meaning and responsibility in life are at the center of life from an existential point of view. Therefore, a hypothetical model was tested to examine the relationships between basic psychological needs (autonomy, competence, relatedness), online gaming addiction, responsibility, and meaning in life. Data were collected from a sample of 546 participants. Mediation analysis was conducted, and the results indicated that basic psychological needs, online gaming addiction, responsibility, and meaning in life had significant negative and positive relationships. The findings indicated that responsibility and meaning in life had a serial mediating effect in the relationship between basic psychological needs and online gaming addiction. The findings also showed that the inverse relationship between online gaming addiction and basic psychological needs was at least partially explained by meaning in life and responsibility. The results of the present study are of great importance and suggest that interventions to satisfy the basic psychological needs of adolescents may help prevent online gaming addiction.

## Introduction

Technological addictions have become an area of increasing research interest and are conceptualized as non-chemical (i.e., behavioral) addictions (Kuss & Billieux, 2017). Moreover, they can be engaged in actively or passively (Widyanto & Griffiths, 2006). For example, television addiction is a passive technological addiction, whereas smartphone addiction and Internet addiction are active technological addictions (Griffiths, 2017). Online addictions have increased rapidly due to the increased use of smartphones, tablets, and laptops. Furthermore, overuse of the Internet has been conceptualized in a number of different ways, including problematic Internet use (Aboujaoude et al., 2006; Young, 2009), excessive Internet use (Choi et al., 2009; Lee et al., 2008), and Internet addiction (Griffiths, 2017) with some considering it to be an impulsive disorder (Young & Rodgers, 2009). In addition, online gaming addiction, which is another addiction associated with the Internet, is defined by the American Psychiatric Association (APA, 2013) as the consistent and prolonged use of the Internet to play videogames, frequently with other gamers, that causes disruption and clinically impairs several aspects of a person’s life (e.g., personal relationships, occupation and/or education). Key characteristics of online gaming addiction are individuals obsessively playing online videogames to the point of neglecting everything else in their lives, which leads to social and/or psychological disorders in such individuals (Ates et al., 2018; Batmaz & Çelik, 2021).

Previous studies have indicated various variables that predict and/or are associated with gaming addiction, including attention-deficit/hyperactivity disorder (ADHD), obsessive-compulsive disorder (OCD), anxiety and depression (Andreassen et al., 2016), social anxiety (Karaca et al., 2020), low self-esteem (Kim et al., 2022), inter-personal competence (Lee et al., 2019), relationship problems and relationship problems, and hostile family environment (Sela et al., 2020). In addition, social skill deficits (Mun & Lee, 2022), social and psychological isolation (Young, 2009), perceived stress (Rajab et al., 2020), suicidality (Erevik et al., 2022), and aggressive behaviors (McInroy & Mishna, 2017) have been reported among individuals who develop gaming addiction.

Although online gaming meets the various needs of individuals, when the behavior turns into an addiction, it leads to adverse effects on individuals, especially adolescents, where it can impair their mental health (Batmaz et al., 2020; Purwaningsih & Nurmala, 2021). Among adolescents, online gaming addiction has been reported to disrupt mental health, increase depression, anxiety, and psychoticism, disrupt family relationships (De Pasquale et al., 2020), lower quality of life (Beranuy et al., 2020), increase social phobia (Wei et al., 2012), lower school performance, and improve sleep deprivation (Chamarro et al., 2020; Király et al., 2015). In short, online gaming addiction negatively affects adolescents’ lives in different areas (Griffiths, 2022; Haberlin & Atkin, 2022). Therefore, research is needed to delineate the causes of online gaming addiction, eliminate its adverse effects, and implement necessary treatment.

Although many studies have been conducted examining online game addiction among adolescents (see Rosendo-Rios et al., 2022) for a recent review of studies), there are few studies examining the relationship between basic psychological needs and online game addiction (Bekir & Celik, 2019). In the present study, it is posited that basic psychological needs could be predictors due to the relationship with gaming disorders and problematic gaming (Allen & Anderson, 2018; Liu et al., 2021; Yu et al., 2015). When basic psychological needs are not met, it pushes individuals to exhibit maladaptive behavioral reactions (i.e., online gaming addiction) (Bekir & Çelik, 2019). In addition, few studies have addressed the relationship between responsibility and meaning in life and online game addiction (Arslan, 2021; Kaya, 2021). Moreover, no study has ever examined the mediating role of responsibility and meaning in life in the relationship between basic psychological needs and online game addiction. For these reasons, the present study examined the mediating roles of responsibility and meaning in life in explaining the relationship between basic psychological needs and online game addiction.

## Online Gaming Addiction and Basic Psychological Needs

Self-determination theory is a well-established motivational theory comprising six mini-theories (Ryan & Deci, 2017). One of these mini-theories is the Basic Psychological Needs Theory (BPNT), which claims that the satisfaction of basic psychological needs is associated with better health and greater psychological well-being (Ryan & Deci, 2000). Basic psychological needs are requirements for psychological development, integrity, and well-being (Deci & Ryan, 2000). In contrast to the often-frustrating real world, videogames are designed to satisfy all three psychological needs (i.e., autonomy, competence, and relatedness) (Rigby & Ryan, 2011). Satisfaction of the needs for competence, autonomy, and relatedness can explain large amounts of the variance in game enjoyment (Rigby & Ryan, 2011; Tamborini et al., 2011). Online gaming can fulfill the (i) need for relatedness by directing players to social relationships with real or fictional characters, (ii) need for autonomy by giving them management and control within the game, and (iii) need for competence by making them feel successful in playing challenging videogames (Allen & Anderson, 2018).

Individuals addicted to videogames need novelty seeking, socialization, competition, and/or entertainment (Hussain et al., 2012; Larrieu et al., 2022). Studies have shown that gaming addiction is related to basic needs (Billieux et al., 2015) and psychological needs such as success, independence, fun, and respect (Herodotou et al., 2012). The increasing demand for playing videogames shows that adolescents try to satisfy some of their psychological needs via the Internet (Shen et al., 2013; Turan, 2021). One longitudinal study found that problematic online gaming and satisfaction of basic psychological needs were positively associated (Yu et al., 2015). It has also been reported that adolescents whose basic psychological needs were not met and whose perceived social support was low had high levels of gaming addiction (Yıldırım & Zeren, 2021). In this context, some studies claim that online games are tools for satisfying basic psychological needs (Oliver et al., 2016). However, studies have shown that the low level of basic psychological need satisfaction in real life can be met with high need satisfaction in online gaming, which leads to addiction for a small minority (Kardefelt-Winther, 2014; Rigby & Ryan, 2017; Wu et al., 2013). Based on the aforementioned literature, it was expected that there would be a significant negative relationship between basic psychological needs derived from real-life and online gaming addiction.

### Online Gaming Addiction and Meaning in Life

The debate about the meaning in life has been ongoing for years (Yalom, 2020). Because there are many definitions of meaning in life, making a standard definition of meaning in life has been difficult (King & Hicks, 2021; Park, 2010). Meaning in life is a multifaceted construct conceptualized in various ways that address the value and purpose of life, meaningful life goals, and sometimes spirituality (Jim et al., 2006). According to Ryff (1989), meaning in life is a sign of a sense of direction, goals, and well-being. Frankl (2009) states that meaning in life differs from individual to individual, day to day, and hour to hour. Many studies have been conducted regarding meaning in life and concepts in the literature. For instance, some of these studies assert that meaning in life increases happiness (Debats et al., 1993) and life satisfaction (Yıkılmaz & Demir Güdül, 2015) and that the presence of meaning in life positively affects psychological health (Bailey & Phillips, 2016) and has a high level of meaning that can lower the incidence of depression (Mascaro & Rosen, 2005).

Similar to the aforementioned studies, adolescents’ having meaning in life can protect them from problematic behaviors such as substance abuse and eating disorders (Brassai et al., 2011; Shek et al., 2019). Adolescence is a period of seeking identity (Erikson, 1968) and decision-making (Marcia, 1980). Steger et al., (2006) pointed out that adolescents’ experience of seeking meaning in life or having a meaning in life may be determinative for successful identity development. However, considering that questioning the meaning in life results from the search for identity, it could be speculated that adolescents who constantly play online videogames will be far from such a search. Although studies have shown that adolescents search for identity in while online gaming (Monacis et al., 2017; Subrahmanyam & Šmahel, 2011; Tanhan & Özlem, 2015), it has been reported that excessive online gaming can also make this exploration more maladaptive, and this may lead to online gaming addiction (King & Delfabbro, 2014; Kokkini et al., 2022). One study reported that as gaming addiction decreases among adolescents, the level of meaning in life increases (Kaya, 2021). In general, it is expected in the present study that the existence of meaning in life in among adolescents will reduce online gaming addiction.

### Online Gaming Addiction and Responsibility

One of the characteristic features of online gaming addiction is that individuals spend their time playing online games by procrastinating and/or not doing their daily work (Thatcheret al., 2008). According to the fifth edition of the *Diagnostic and Statistical Manual of Mental Disorders* (DSM-5) criteria, one criterion for Internet gaming disorder is that individuals continue to play online games despite being aware of psychosocial problems (American Psychiatric Association, 2013). Here, individuals fail to engage in important day-to-day responsibilities and play online games instead. Similarly, it has been shown that online gaming addicts jeopardize or lose their job, education, and/or career opportunities to play online games (Kardefelt-Winther, 2014). Time spent playing games instead of engaging in life’s more important tasks can be viewed as a lack of responsibility by individuals themselves, their families, and/or friends (Wartberg et al., 2017; Zhang et al., 2019).

Responsibility consists of three elements: accountability, liability, and imputability (Robinson, 2009). Imputability refers to individuals being responsible for their actions and decisions, accountability refers to fulfilling contractual expectations, and liability refers to assuming a moral responsibility without a contract (Holdorf & Greenwald, 2018). The concept of responsibility therapy is defined as the ability of individuals to meet their own needs while allowing others around them to meet their needs (Corey, 2015). Being conscious of responsibility means that individuals are aware of themselves and their feelings, thoughts, and pain (Yalom, 2020). Dökmen (2019) defines it as a responsibility to accept the consequences on others of what an individual does or does not do based on his thoughts.

In addition, it is discussed in the literature under two dimensions: emotion (Berkowitz & Daniels, 1963; Özen, 2013) and behavior (Glasser, 2005; Taylı, 2006). Individuals with a sense of responsibility have characteristics such as acting with awareness of their own and others’ rights, respecting others, and attempting to fulfill their responsibilities (Özen, 2011; Yough et al., 2022). On the other hand, individuals who do not have a sense of responsibility make themselves and others feel worthless while living without a plan or program (Cüceloğlu, 2015). Studies have shown that a low sense of responsibility can lead to aggression, lying, and avoidance of responsibility, while a high level of responsibility can trigger perfectionism, leading to anxiety, depression, and obsessive-compulsive disorders (Taylı, 2013; Wang et al., 2022).

The behavior of responsibility, the second sub-dimension of responsibility (Yalom, 2020), means that individuals can take responsibility by bearing the consequences of their behavior without attributing it to someone else (Douglass, 2001; Shahzadi et al., 2022). It has a function that improves positive activities and prevents harmful activities (Kesici, 2018). For example, individuals who act responsibly are respected by society and avoid punishment (Douglass, 2001). On the other hand, during adolescence, when serious responsibilities begin to be undertaken, a minority of individuals may move away from social life due to gaming addiction. Because of this situation, other people in the individual’s social life (e.g., family and friends) become unimportant to adolescents with low awareness of responsibility. Recent studies have observed that adolescents who excessively play videogames have difficulty fulfilling their responsibilities (Dinçer & Kolan, 2020; Doğan & Pamuk, 2022). In the present study, it was expected that adolescents with higher levels of responsibility would be less addicted to online gaming (i.e., an inverse relationship).

### Basic Psychological Needs, Meaning in Life, Responsibility, and Online Game Addiction

Basic Psychological Needs Theory (BPNT) focuses on the satisfaction and frustration of psychological needs and argues that these needs significantly impact individuals’ psychological health and well-being (Ryan & Deci, 2000). Lack of fulfillment of basic psychological needs leads to negative consequences (e.g., depression, stress, and addiction) (Cantarero et al., 2021; Levine et al., 2022; Orkibi & Ronen, 2017; Xiao & Zheng, 2022). However, satisfying these needs is associated with positive outcomes such as general self-efficacy (İhsan et al., 2011), mental resilience (Kilinç & Gürer, 2019), subjective well-being (Akbağ & Ümmet, 2018), and obtaining meaning in life (Çelik & Gazioğlu, 2017). Furthermore, Weinstein et al. (2012) suggested that the search for meaning increased significantly when these needs were satisfied. Individuals whose needs are fulfilled are more prone to seek meaning in their life and, therefore, to experience meaning in their life, whereas individuals whose needs are not fulfilled experience a sense of meaninglessness (Eakman, 2013). According to Steger (2006), although individuals continue to search for meaning in one area of their lives, they may have meaning in a different area of their life. Meaning in life is defined as the purpose and importance of the life that individuals derive from their experiences (Baumeister & Vohs, 2002; Steger et al., 2006). Frankl (1969) posited that to achieve the meaning of life, an individual must take responsibility for realizing their potential, even at a young age. Therefore, a meaningful life requires individuals taking responsibility for themselves and others.

Responsibility refers to the individual’s sense of duty toward family, friends, and society (Geçtan, 2006), and can be examined in personal and social dimensions (Arslan & Wong, 2022). Personal responsibility means that an individual is accountable to themselves and to the needs or well-being of others (Ruyter, 2002). It also emphasizes self-responsibility by representing the individual’s behaviors and choices that can affect themselves and others (Mergler & Shield, 2016). Social responsibility relates to values that support individuals’ moral and prosocial behavior (Wray-Lake & Syvertsen, 2011). It includes decisions and actions that benefit others and society (Martins et al., 2015). Moreover, it is an important source of support in strengthening individuals’ mental health and improving their life skills (Martins et al., 2017) as well as coping with addictions (Amini et al., 2020). Therefore, individuals’ personal and social responsibility can protect them against negative situations such as developing addictions (e.g., online gaming addiction) (Chiou & Wan, 2007).

Online games allow individuals to meet other players, have fun, achieve status, and obtain financial benefits (Ballabio et al., 2017; Columb et al., 2022). In addition, escaping from the problems of real life, even temporarily, and achieving relaxation are among the benefits that individuals gain through gaming (Yee, 2006). Consequently, online gaming can lead individuals to play online games frequently and for long periods of time, which in turn can lead to the risk of addiction (Luciana, 2010; Sachdeva & Verma, 2015). The 11th revision of the *International Classification of Diseases* (ICD-11) characterized gaming disorder as a repetitive or persistent pattern of gaming behavior (World Health Organization, 2019). Individuals that are affected by online gaming addiction have also been reported to experience problems with interpersonal relationships (Wongpakaran et al., 2021), occupation (Lelonek-Kuleta et al., 2021), and health (Chan et al., 2022). As such, online gaming addiction can lead to situations that threaten the lives and functionality of individuals through the process and its consequences.

### The Present Study

The present study was framed according to self-determination and existentialist positive psychology theories. Self-determination theory (SDT) suggests that the non-satisfaction or inhibition of basic psychological needs can lead to negative consequences (i.e., online gaming addiction). In addition, it emphasizes that behaviors emerge from the individual’s beliefs, meaning, and value judgments rather than external factors (i.e., social norms and group pressure). According to the SDT, need (autonomy, competence, and relatedness) predicts meaning in life (Eakman, 2013). Moreover, in a longitudinal study based on SDT, individuals whose basic psychological needs were fulfilled had increased meaning in life (Zhang et al., 2022). In addition, the existentialist theory of positive psychology suggests that the meaning in life, which individuals create themselves, can be sustained through responsibility. Individuals having responsibility can also enable them to lead a meaningful life (Arslan & Yıldırım, 2021; Wong, 2019). According to Wong (2010), meaning consists of the components of purpose, understanding, responsibility, and enjoyment (PURE). In addition to responsibility being one of the basic concepts that constitute meaning, the search for meaning in life continues intensely during adolescence (Steger, 2012). This is especially the case for adolescents who begin to question people and the world deeply, having a meaningful life can protect them from behavioral addictions (Qiu et al., 2022; Zhao et al., 2020). Considering the role of responsibility and meaning in the life of adolescents, it is important to examine online game addiction, which may be affected by basic psychological needs. Therefore, a serial mediation model was determined based on the assumptions of self-determination and existential positive psychotherapy theory.

In addition to the aforementioned theoretical framework, studies have shown that unfulfilled basic psychological needs are predictors of online gaming addiction (Allen & Anderson, 2018; Liang et al., 2021; Mills & Allen, 2020; Yu et al., 2015). However, studies conducted with adolescents have found a relationship between online gaming addiction and responsibility and meaning in life (Doğan & Pamuk, 2022; Kaya, 2021). In the present study, which also considers the different dynamics in online gaming addiction, a new model is proposed to examine the relationship between basic psychological needs and online gaming addiction through responsibility and meaning in life. In this context, the present study assessed whether basic psychological needs (i.e., autonomy, relatedness, competence) affect the relationship between online gaming addiction, meaning in life, and responsibility among adolescents. Four research questions were investigated: Do basic psychological needs predict online gaming addiction? (RQ1); Does the level of responsibility have a mediating effect on the relationship between basic psychological needs and online game addiction? (RQ2); Does meaning in life have a mediating effect on the relationship between basic psychological needs and online game addiction? (RQ3); Do responsibility and meaning in life have a serial mediating effect on the relationship between basic psychological needs and online game addiction? (RQ4).

## Method

### Participants

Power analysis was performed via the G* Power 3.1.9.7 program to determine the sample size required for the present study. For this purpose, at the conventional significance level of 0.05 and power at 0.80, a small effect size is determined as *r* = 0.20 (Cohen, 2013). As a result of the analysis, it was determined that the required sample size was 395. The sample in the present study comprised 546 individuals (393 females and 153 males). The participants ranged from 15 to 18 years old, with a mean age of 16.25 years (SD ± 0.82). Just below half the sample of the participants were in the 9th grade (*n*=252; 46.2%), 156 were in the 10th grade (28.6%), 74 were in the 11th grade (13.6%), and 64 were in the 12th grade (11.7%). Over one-third of the sample self-reported their socioeconomic status (SES) as being low (*n*=210; 38.5%), 224 reported it as being medium (41.0%), and 112 reported it as being high (20.5%). Participants stated that they played videogames 3.56 h daily on average (SD ± 3.12). The number of devices they used to play online videogames was 2.09 (SD ± 0.96).

### Measures

#### Basic Psychological Needs Scale (BPNS)

The 21-item BPNS (Deci & Ryan, 2000; Turkish version: Kesici et al., 2003) was used to assess basic psychological needs. The scale consists of three subscales: (i) autonomy (AU), (ii) competence (CMP), and (iii) relatedness (RLT). The scale has 21 items that tap into the satisfaction of autonomy (e.g., “I feel free to decide how to live my life”), relatedness (e.g., “There aren’t many people in my life that I feel close to”), and competence (e.g., “The people I know say that I am successful in what I do”) which are rated on five-point Likert scale from 1 (*strongly disagree*) to 5 (*strongly agree*). The higher the score, the greater fulfillment of autonomy, competence, and relatedness. In the present study, the scale’s internal reliabilities for the need for autonomy were α=.76, McDonald’s ω= 76; the need for competence were α =.67, McDonald’s ω= 68; and the need for relatedness were α =.82, McDonald’s ω= 83.

#### Meaning in Life Questionnaire Scale (MILQS)

The 10-item MILQS (Steger et al., 2006; Turkish version: Demirbaş-Çelik and İşmen-Gazioğlu, 2015) was used to assess meaning in life. Items (e.g., “I’m always looking for my life’s purpose”) are rated on seven-point Likert scale from 1 (*definitely disagree*) to 7 (*definitely agree*). The total score ranges between 10 and 70. The higher the score, the higher the individual’s level of search for meaning in life. In the present study, the internal reliability for the existence of meaning in life was α=.85 and for seeking meaning in life was α=.82. For the overall scale, Cronbach’s α was .67, and McDonald’s ω was .72.

#### Sense of Responsibility and Behavior Scale (SRBS)

The 18-item SRBS (Özen, 2013) was used to assess responsibility. Items (e.g., “I feel responsible for being a member of charitable organizations”) are rated on four-point scale ranging from 1 (*never*) to 4 (*always*). The total score ranges between 18 and 72. The higher the score, the greater the level of responsibility. The SRBS consists of two subscales and each can be used separately. The sense of responsibility sub-dimension was used in the present study. For this sub-dimension, Cronbach’s α was .86, and McDonald’s ω was .87.

#### Online Game Addiction Scale (OGAS)

The 21-item OGAS (Başol & Kaya, 2018) was used to assess online gaming addiction. Items (e.g., “My friendships were damaged/broken due to online games”) are rated on a five-point Likert scale ranging from 1 (*absolutely disagree*) to 5 (*absolutely agree*). The total score ranges between 21 and 105 points. The higher the score, the greater the risk of online gaming addiction. In the present study, Cronbach’s α was .88, and McDonald’s ω was .89.

### Procedure and Ethics

Participants were selected from three different high schools in Turkey in the cities of Ağrı, Karabük, and Siirt. The schools were informed about the purpose and duration of the study. The researchers visited the schools, and informed consent forms were distributed. Written informed consent forms were obtained from the legal guardians or parents of the adolescents who volunteered to participate in the study. The purpose of the study was explained to the participants. The eligibility criteria for participation in the study were being an adolescent and being an individual who played (or used to play) one or more online videogames. An online link to the survey was sent to the participants, and each participant was allowed to complete the survey only once. All data were collected using *Google Forms* in the classroom. Participants were reminded that they might stop answering at any stage of the survey process if they wanted to. Participants were asked not to provide personal information to ensure anonymity and confidentiality. Ethics committee approval of this research was obtained from Ağrı İbrahim Çeçen University (reference number: 110), and every research stage was carried out in accordance with the Declaration of Helsinki.

### Data Analysis

All analyses were carried out using SPSS version 26, Hayes’ (2018) PROCESS Macro (version 3), and G* Power 3.1.9.7 programs. Before starting the analysis, the necessary assumptions to perform the analysis were tested. The kurtosis and skewness values were examined to understand whether the assumptions required for the prerequisites of parametric tests were met. The skewness and kurtosis values for a normal distribution have acceptable threshold values if they are ±2 (George, 2010). There were no assumption violations in the research data. In addition, it was found that the correlation between the study variables was not high. The correlations ranged between .17 and .63 (*p*<.001). The research variables were also examined to ensure there were no multicollinearity issues. When the tolerance, variance inflation factor (VIF), and confidence interval (CI) values were examined, these values were all within acceptable limits. It was determined that VIF was between 1.12 and 1.48, the tolerance value was between .67 and .89, and CI was between 7.21 and 17.88. The limit values required to avoid multicollinearity problems are more than 0.20 for the tolerance value, less than 10 for the VIF value, and less than 30 for the CI value (Albayrak, 2005; Büyüköztürk, 2016; Şata, 2020). Consequently, no multicollinearity problems were detected. Mahalanobis distance values were examined to determine whether there were outliers in the sample. A total of 21 outliers were identified in the dataset. These outliers were excluded from the analysis, meaning the final sample size was 546. SPSS PROCESS macro was utilized to conduct mediation analyses (Hayes, 2018). The bootstrapping method was employed with 5000 resampling and 95% confidence intervals (CIs) to test the significance of the mediating pathways. An effect is deemed significant if the confidence interval does not contain zero (Preacher & Hayes, 2008).

## Results

Table 1 shows the correlations between all the main variables in the study (basic psychological needs (autonomy, competence, relatedness), online gaming addiction, responsibility, and meaning in life). Pearson correlations indicated that all variables were significantly (albeit moderately and weakly) related.

**Table 1:** Descriptive statistics and bivariate correlations among variables in the total sample (*N* = 546)

	Autonomy	Competence	Relatedness	Online gaming addiction	Responsibility	Meaning in life
Autonomy	_-_					
Competence	^.61**^	^-^				
Relatedness	^.63**^	^.52**^	^-^			
Online gaming addiction	^-.23**^	^-.18**^	^-.20**^	^-^		
Responsibility	^.25**^	^.22**^	^.30**^	^-.30**^	^-^	
Meaning in life	^.27**^	^.35**^	^.27**^	^-.19**^	^.28**^	^-^
Mean	22.57	18.97	27.92	47.56	59.63	49.87
Std. deviation	5.32	4.26	6.28	15.49	7.60	9.31
Skewness	^-^0.40	^-^0.31	^-^0.45	0.23	^-^0.89	^-^0.17
Kurtosis	0.34	0.23	0.10	^-^0.56	1.6	0.04

**p < 0.001

### Serial Multiple Mediational Analyses—Modeling Data

Table 2, Table 3, and Table 4 show the results of the serial mediation analysis. First, there was a direct effect of autonomy on online gaming addiction (*β*=−.67, *p*<.001). Moreover, the relationship between competence and online gaming addiction was examined. There was a direct effect of competence on online gaming addiction (*β*=−.63, *p*<*.*001). When the relationship between relatedness, the last of the basic psychological needs, and online gaming addiction was examined, there was a direct effect of relatedness on online gaming addiction (*β*=−.48, *p*<*.*001). There was also a significant indirect effect of autonomy on online gaming addiction via responsibility (indirect effect=−.12, SE=.02, *95%* CI= [−.20, −.06]). Also, the indirect effect of competence on online gaming addiction via responsibility was significant (indirect effect=−.19, SE=.02, *95%* CI= [−.31, −.10]). Lastly, the indirect effect of relatedness on online gaming addiction via responsibility was significant (indirect effect=−.17, SE=.01, *95%* CI= [−.26, −.10]).

**Table 2:** The indirect effect of basic psychological needs autonomy on online gaming addiction via meaning in life and responsibility

Path	_Confficient_	95% Confidence interval		Decision
		Lower limit	Upper limit	
Autonomy → Meaning in life → Online gaming addiction	− 0.07	− 0.14	− 0.00	Supported
Autonomy → Responsibility → Online gaming addiction	− 0.12	− 0.20	− 0.06	Supported
Autonomy → Meaning in life → Responsibility → Online gaming addiction	− 0.04	− 0.07	− 0.01	Supported
Total effect	− 0.67	− 0.91	− 0.43	
Direct effect	− 0.44	− 0.68	− 0.20	
Total indirect effect	− 0.23	− 0.34	− 0.13

**Table 3:** The indirect effect of competence on online gaming addiction via meaning in life and responsibility

Path	_Coefficient_	95% Confidence interval		Decision
		Lower limit	Upper limit	
Competence → Meaning in life → Online gaming addiction	− 0.11	− 0.22	− 0.00	Supported
Competence → Responsibility → Online gaming addiction	− 0.19	− 0.31	− 0.10	Supported
Competence → Meaning in life → Responsibility → Online gaming addiction	− 0.02	− 0.06	− 0.01	Supported
Total effect	− 0.63	− 0.93	− 0.33	
Direct effect	− 0.31	− 0.63	− 0.01	
Total indirect effect	− 0.32	− 0.48	− 0.17

**Table 4:** The indirect effect of relatedness on online gaming addiction via meaning in life and responsibility

Path	_Coefficient_	95% Confidence interval		Decision
		Lower limit	Upper limit	
Relatedness → Meaning in life → Online gaming addiction	− 0.05	− 0.10	− 0.01	Supported
Relatedness → Responsibility → Online gaming addiction	− 0.17	− 0.26	− 0.10	Supported
Relatedness → Meaning in life → Responsibility → Online gaming addiction	− 0.02	− 0.04	− 0.00	Supported
Total effect	− 0.48	− 0.68	− 0.27	
Direct effect	− 0.24	− 0.45	− 0.03	
Total indirect effect	− 0.24	− 0.34	− 0.14	

When indirect effects were examined, there was a significant indirect effect of autonomy on online gaming addiction via meaning in life (indirect effect=−.07, SE=.02, *95%* CI= [−.14, −.00]). Also, the indirect effect of competence on online gaming addiction via meaning in life was significant (indirect effect=−.11, SE=.02, *95%* CI= [−.22, −.00]). Lastly, the indirect effect of relatedness on online gaming addiction via meaning in life was significant (indirect effect=−.05, SE=.01, *95%* CI= [−.10, −.01]).

Moreover, the indirect effects of autonomy on online gaming addiction via meaning in life and responsibility were tested. The effect was significant (testing serial multiple mediation; effect=−.04 SE=.01, 95% CI= [−.07, −.01]). Also, the indirect effects of competence on online gaming addiction via meaning in life and responsibility were tested. The effect was significant (testing serial multiple mediation; effect=−.02 SE=.01, 95% CI= [−.04, −.00]). Moreover, the indirect effects of relatedness on online gaming addiction via meaning in life and responsibility were tested. The effect was significant (testing serial multiple mediation; effect=−.02 SE=.01, 95% CI= [−.06, −.01]). In the relationship between basic psychological needs (autonomy, competence, relatedness) and online gaming addiction, meaning in life and responsibility had serial mediating effects.

**Fig. 1:**
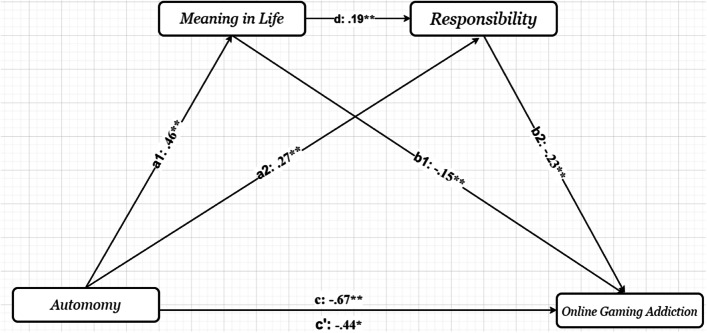
The results of the serial multiple mediational models

The results indicated that autonomy predicted online gaming addiction. When autonomy was entered as the predictor, it significantly predicted online gaming addiction (*β* = −0.67, t = −5.58, *p* < .001), and accounted for 5.4% of the variance in the model. Figure 1 shows the regression coefficients of the mediation model. The indirect path mediated by responsibility (*β* =−.12, 95% CI= [−.20, −.06]) produced a higher change in variance than the indirect path mediated by meaning in life (*β* =−.07, 95% CI= [−.14, −.00]) in the relationship between relatedness and online gaming addiction (see Table 2). Therefore, responsibility appeared to have a higher effect than meaning in life. Autonomy predicted a higher level of meaning in life. It also predicted a higher level of responsibility. Higher meaning in life was associated with a higher level of responsibility. Higher level of responsibility was associated with lower online gaming addiction. Consequently, the results indicated that the relationship between autonomy and online gaming addiction was partially mediated by meaning in life and responsibility (see Fig. 1).

**Fig. 2:**
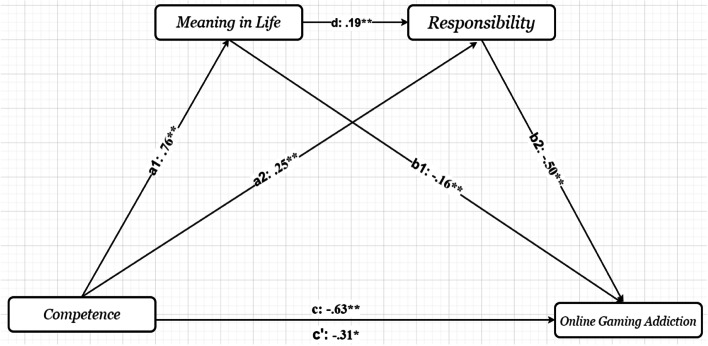
The results of the serial multiple mediational models

It was also found that competence predicted online gaming addiction. There was also an indirect relationship between competence and online gaming addiction (*β* = −0.64, t = −4.13, *p* < .001), accounting for 4.7% of the variance in the model. Competence predicted meaning and responsibility in life. The indirect path mediated by responsibility (*β* =−.19, 95% CI= [−.31, −.10]) produced a higher change in variance than the indirect path mediated by meaning in life (*β* =−.11, 95% CI= [−.22, −.00]) in the relationship between competence and online gaming addiction Furthermore, the relationship between competence and online gaming addiction was mediated by meaning in life and responsibility separately (see Table 3). The results also showed that meaning in life and responsibility had serial mediation effects in the relationship between competence and online gaming addiction (see Fig. 2).

**Fig. 3:**
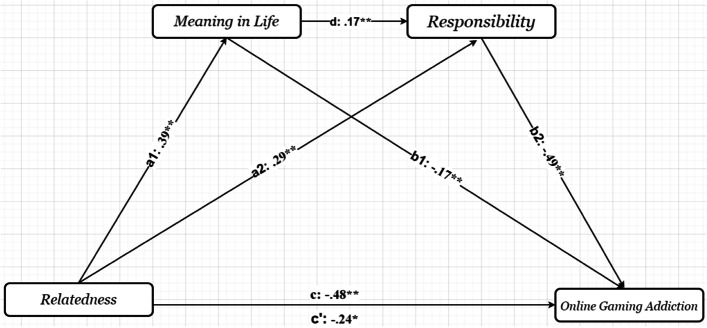
The results of the serial multiple mediational models

Lastly, the results indicated that relatedness predicted online gaming addiction (*β* = −0.48, t = −4.63, *p* < .001). When relatedness was included in the model, it was found that it accounted for 3.8% of the variance. Moreover, there was also an indirect relationship between relatedness and online gaming addiction. When the indirect effects are examined, the indirect path mediated by responsibility (*β* =−.17, 95% CI= [−.26, −.10]) produced a higher change in variance than the indirect path mediated by meaning in life (*β* =−.05, 95% CI= [−.10, −.01]) in the relationship between relatedness and online gaming addiction (see Table 4). The results suggested that the relationship between relatedness and online gaming addiction was partially mediated by meaning in life and responsibility (see Fig. 3).

## Discussion

In self-determination theory (SDT), basic psychological needs comprise autonomy, competence, and relatedness. In SDT (Deci & Ryan, 2000), basic psychological needs are expressed as essential psychological nutrients for psychological development, integrity, and well-being. Negative psychological consequences occur when requirements are not met, neglected, or prevented (Deci & Ryan, 2000). If individuals cannot satisfy a basic need, they engage in activities that give pleasure to individuals momentarily, even if they do not satisfy them (Antunes et al., 2020; Deci & Ryan, 2011). One of these activities is online gaming, which has an incredibly interactive structure. At the same time, online videogames are appreciated because they create an environment where both the need for relatedness and autonomy are met in the virtual world. Individuals naturally seek new challenges to experience a sense of efficacy even when no external rewards (e.g., money) are earned (Dindar, 2018; Matsumoto, 2009). The fact that online games have a reward mechanism is suitable for activating feelings of competence among individuals. It is thought that adolescents tend to meet their basic psychological needs (need for autonomy, competence, and relatedness) that they cannot fully meet from their parents or close friends through online gaming.

The present study examined the mediating role of meaning in life and the level of responsibility in the relationship between online gaming addiction and basic psychological needs (i.e., autonomy, competence, and relatedness) among adolescents. Findings showed that autonomy predicted online game addiction. In other words, autonomy had significant negative effect on online game addiction. Considering that addiction is related to reduced autonomy (Amatem, 2008), it can be said that the finding is compatible with the literature. However, there is a study in which there was a negative relationship between the need for autonomy and digital game addiction among adolescents (Dursun and Çapan, 2018), which supports the research finding. On the contrary, there is a study in which autonomy and online game addiction had significant positive relationships (Bekir and Çelik, 2019). Similarly, it is known that the need for autonomy has a positive relationship with social media addiction (Young-Ju et al., 2018) and a negative relationship with Internet addiction (Piri et al., 2018; Zeren & Can, 2019). These studies, which have obtained different results, make the relationship between the need for autonomy and digital addictions open to discussion but also show that further research is needed.

According to the present study’s findings, it was found that relatedness and competence, as well as autonomy, predicted online gaming addiction. Studies have shown that competence and relatedness have significant relationships with online gaming addiction (Bekir and Çelik, 2019; Dursun and Çapan, 2018). In addition, research has shown that relatedness has a negative relationship with short-form video addiction (Yang et al., 2022), and relatedness dissatisfaction positively correlates with Internet gaming disorder (Hui et al., 2019). Moreover, significant negative relationships have been found between competence and smartphone addiction (Gao et al., 2022; Sun et al., 2020) and Internet addiction (Zeren & Can, 2019; Canoğulları, 2014). Based on these results concerning technological addictions, it can be said that the literature findings and the results of the present study are compatible.

The tendency of individuals to play online videogames may be to meet their autonomy, competence, and relatedness needs (Ryan et al., 2006). In addition, when basic psychological needs are prevented, technological addictions (gaming addiction, smartphone addiction, social network addiction, and Internet addiction) increase (Gugliandolo et al., 2020). This may be the compensation for unmet basic psychological needs through addiction (Kuss et al., 2017; Mills et al., 2018). Therefore, fulfilling basic psychological needs in real life and eliminating the problems that prevent this satisfaction can be a protective factor against online gaming addiction.

Another finding of the present study was that the level of responsibility hads a mediating effect on the relationship between basic psychological needs and online gaming addiction. However, there was a positive and significant relationship between basic psychological needs and responsibility. In contrast, a significant negative relationship was found between responsibility and online gaming addiction. Considering that the components of responsibility (accountability, liability, and imputability) in Robinson’s (2009) definition appear less important in online environments, it is assumed that adolescents who are addicted to online gaming experience less sense of responsibility. A recent study found that a higher level of responsibility significantly predicted online gaming addiction, whereas a lower level of responsibility negatively affected online gaming addiction (Kesici, 2020).

Research conducted by Arslan (2021) found that secondary school students’ sense of responsibility and behavior had a crucial predictive role in online gaming addiction. Another study reported a significant negative relationship between the students’ videogame addiction and their personal and social responsibility behavior (Dinçer & Kolan, 2020). Based on previous studies and the results of the present study, it is thought that increasing the level of responsibility of secondary and high school students would reduce gaming addiction. Adolescents whose level of responsibility increases are also more likely to engage in responsible behavior. This is supported by studies in the literature that physical education and sports play an essential role in helping adolescents acquire responsible behavior (Bayraktar et al., 2016; Bugdayci, 2019; Tazegül, 2014). These studies’ results are considered necessary regarding online gaming addiction because such behavior leads to a sedentary lifestyle (Cómez-Mármol et al., 2017).

Findings indicated that meaning in life had a mediating effect on the relationship between basic psychological needs and online gaming addiction. However, there was a positive and significant relationship between basic psychological needs and meaning in life. In contrast, a significant negative relationship between meaning in life and online gaming addiction was found. These findings demonstrate the importance of meaning in life in preventing online gaming addiction among adolescents. A study by Kaya (2021) on adolescent online gaming addiction found that as the level of online gaming addiction decreased, the level of meaning in life increased. These results suggest that meaning in life affects online gaming addiction as a cause and consequence. Considering that having a meaningful life increases resilience (Batmaz et al., 2021; Doğrusever et al., 2022), low resilience increases gaming addiction (Canale et al., 2019), and gaming addiction reduces happiness (Kaya, 2021; Turan, 2021), meaning in life seems to be an essential variable that can affect gaming addiction.

What makes the present study unique to the online gaming addiction literature is that responsibility and meaning in life had a serial mediating effect on the relationship between basic psychological needs and online gaming addiction. In other words, the results indicated that the relationship between relatedness, competence, and autonomy with online gaming addiction was partially mediated by meaning in life and responsibility. This finding suggests that the need for autonomy, competence, and relatedness increases the level of meaning in life, which in turn reduces online game addiction. Similarly, online game addiction can decrease as the need for autonomy, competence, and relatedness increases the level of responsibility. In addition, based on the serial mediation effect, it suggests that meeting the need for autonomy, competence, and relatedness can reduce adolescents’ online game addiction by increasing their meaning in life and their level of responsibility.

To the best of the authors’ knowledge, the present study is the first to examine the mediating role of responsibility and meaning in life between basic psychological needs and online gaming addiction. The associations between these variables provide greater understanding and knowledge concerning online gaming addiction and provide additional insight into the significant causes that underlie playing games online (which may be potential factors in the acquisition, development, and maintenance of online gaming addiction among adolescents). Moreover, fulfilling basic psychological needs appears to increase responsibility and meaning in life and reduce susceptibility to online gaming addiction. The findings enrich the literature because it suggests new protective factors that might prevent adolescents from developing online gaming addiction.

The findings offer relevant practical implications for adolescents, educators, families, private and public health institutions, and mental health professionals to assist them in designing addiction prevention strategies and policies. Results also suggest that basic psychological need satisfaction fulfilment in real life plays an important role in the development and maintenance of online gaming addiction among adolescents. Educators, parents, and adolescents could utilize awareness of the factors contributing to online gaming addiction to help them take preventive measures against it. In addition, if adolescents have high levels of responsibility and meaning in life, it may help reduce online game addiction. Considering the findings, it is recommended that mental health professionals provide training and services that increase the level of responsibility among adolescents and enable them to have meaning in their lives to prevent the onset of online gaming addiction. In addition, private and public health institutions should implement training programs to improve the skills of parents, such as digital parenting, to cope with online gaming addiction. This training should also ensure that parents behave with awareness of the basic psychological needs of adolescents in the family and that they gain thoughts and approaches that can add responsibility and meaning in life.

## Limitations

As in all studies, the present study also has some limitations. The first is that the study was cross-sectional. Conducting a cross-sectional study means that causality between the study variables cannot be determined. Second, completing the survey online may have influenced respondents’ responses (with those without home Internet access unable to participate). The online data were also collected during the COVID-19 pandemic. Therefore, adolescents living in isolated environments may have increased their gaming during this period. This unusual situation may have resulted in a lower sense of responsibility and a less meaningful life. This is consistent with the present study’s findings. Another limitation is that the participants were high school students studying in different schools in Turkish provinces, so the findings are not necessarily generalizable to all Turkish schoolchildren. The sample was also limited because it did not include other education levels, such as primary and secondary schools and children from different geographical and cultural regions in Turkey and/or other countries. Future studies are needed with different age groups, such as primary school, secondary school, university students, adults, and various geographical regions in the sample groups (both in and outside Turkey). Such studies are needed to confirm the findings reported here and should include other research designs (e.g., longitudinal studies to determine causality between variables) and other types of data (e.g., qualitative interview data to attain richer data). Another limitation of the present study was that the participant’s responses were self-report and therefore subject to well-established method biases (e.g., social desirability, memory recall).

## Conclusion

The study’s findings indicated that adolescents whose basic psychological needs were met exhibited lower levels of online gaming addiction than adolescents whose basic psychological needs were not met. Consequently, the adverse effects of online gaming addiction may be reduced by interventions that meet adolescents’ basic psychological needs. Similarly, a significant negative relationship was found between responsibility and online gaming addiction. Consequently, it appears that adolescents who fulfill the requirements of individual and social responsibilities (studying, spending time with family, going out with friends, etc.) have greater protection from the more negative effects of online gaming. However, when meaning in life and responsibility are included in the relationship between basic psychological needs and online gaming addiction, the effect of basic psychological needs on online game addiction decreases. This suggests that meaning in life and responsibility have a serial mediating role between basic psychological needs and online gaming addiction.

## Data Availability

The data that support the findings of this study are available from the first author upon reasonable request.
